# Risk Criteria in Hospital Site Selection: A Systematic Review

**DOI:** 10.1371/currents.dis.a6f34643f3cd22c168b8c6f2deeae86d

**Published:** 2017-05-01

**Authors:** Mohammad Javad Moradian, Ali Ardalan, Amir Nejati, Ali Darvishi Boloorani, Ali Akbarisari, Behnaz Rastegarfar

**Affiliations:** Department of Disaster Public Health, School of Public Health, Tehran University of Medical Sciences, Tehran, Iran; Trauma Research Center, Shahid Rajaee (Emtiaz) Trauma Hospital, Shiraz University of Medical Sciences, Shiraz, Iran; Department of Disaster & Emergency Health, National Institute of Health Research, Tehran University of Medical Sciences, Tehran, Iran; Department of Disaster Public Health, School of Public Health, Tehran University of Medical Sciences, Tehran, Iran; Harvard Humanitarian Initiative, Harvard University, Cambridge, MA, USA; Emergency Medicine Research Center, Tehran University of Medical Sciences, Tehran, Iran; Geoinformatics Research Institute, Department of Remote Sensing & GIS, University of Tehran, Tehran, Iran; Department of Health Management and Economics, School of Public Health, Tehran University of Medical Sciences, Tehran, Iran; Department of Disaster and Emergency Health, Tehran University of Medical Sciences, Tehran, Iran

## Abstract

**Introduction:**

Hospitals should be safe and remain functional in emergencies and disasters as it is mentioned in the Sendai Framework. Proper selection of a hospital location has a direct effect on survival of affected population in disasters as well as cost and benefit of the hospital in non-emergency situation. Different studies applied different criteria for Hospital Site Selection (HSS). The present study through a systematic review aimed to find out a categorized criteria list that have been used for (HSS) in the literature.

**Methods:**

In accordance with the PRISMA statement, “PubMed”, “ScienceDirect”, “Google Scholar”, and “Scopus” were searched up to end of 2015. All English Articles that were published in peer-reviewed journals and had discussed site selection criteria for hospitals were included. Out of 41 articles, 15 met the inclusion criteria in which 39 general criteria for HSS were applied. These criteria were categorized in six main groups including cost, demand, environmental, administrative, disaster risk, and “other” concerns through a focus group discussion.

**Results:**

Accordingly, the application percentage of cost, demand, environmental, administrative, disaster risk, and “other” concerns in the articles was 100, 93.3, 53.3, 33.3, 20.0, and 13.3 respectively. The least devoted attention was to disaster risk issues.

**Discussion:**

Few researchers applied risk related criteria for HSS. Further consideration of “risk of hazards” and “burden of diseases” in comprehensive studies, is recommended for HSS to guide the decision makers for building more resilient hospitals. Keywords   Hospital, Site selection, Systematic review, Disaster risk

## Introduction

Hospitals are one of the main elements of social services, and a cornerstone of response to disasters in an acute phase, especially in countless mass casualty incidents. Social service delivery has its roots in the time when humans began living together as a community to meet their needs. Accordingly, health-related services were developed particularly in more centralized populations, when diseases and injuries became one of the most challenges besides food and water^1^.

The percentage of people living in cities is rapidly increasing due to having an easy access to social services. To provide a chance for having equitable access to hospitals a convenient location of establishing the service centers is of great importance^1^. In the decision-making process to establish a new hospital or renovate an old one proper location plays an important role specifically with regard to guaranteeing the profit return on investment. In other words, determining the location of a hospital is an important factor that can affect the cost and benefits^2^. In 2006, Younis et al showed that the geographic location influences the profitability of a hospital i.e. financial performance^3^. Considering the projections related to the increase in urban population to greater than 5 billion by 2025 and its effects on increasing the vulnerability in addition to climate-change related risks, assigning a proper location for medical centers becomes a crucial factor for planners^4^^,^^5^^,^^6^, given its long time impacts^7^. For instance, in a study by Bell (2007) it was indicated that the location of a hospital would have a direct effect on survival in situations such as nuclear attacks^8^. Also Ochi (2014) stated that inappropriate location may cause damages to a hospital due to external hazards such as earthquakes^9^.

On the other hand, Sendai framework emphasizes structural disaster risk prevention and reduction measures as well as the promotion of resilience of new and existing critical infrastructures such as hospitals^10^.

In this regard, there are two main theories used to optimize hospital location. The first is based on the Weberian model, which focuses on a single objective namely the minimum cost or maximum profit. (WU 2007) The second theory has its roots in “behavioral approach” which simultaneously considers several factors to determine the most appropriate location. For instance, Analytic Hierarchy Process (AHP), a Multi-Criteria Decision-Making (MCDM) method, is based on “behavioral approach”^11^.

The present study through a systematic review aims to retrieve a list of disaster risk related criteria applied in hospital site selection (HSS).

## Methods

**Study design**

This study is a descriptive systematic review investigating risk criteria in HSS. The 27-item Preferred Reporting Items for Systematic Review and Meta-analysis statement (PRISMA) 2009 checklist is used as a reference^12^. The study protocol was approved by the Higher Education Council of School of Public Health at Tehran University of Medical Sciences.

**Search methods for identification of studies**

In the present study, “hospital “refers to the legal institution that provides 24-h medical services, including accepting, visiting, admission, and treatment of injured and/or sick individuals^13^^,^^14^. “Site selection” refers to an operational problem solving research in which the researchers will find out the best location that meets the assigned preferences^15^. “Risk” refers to both the probability of an event (hazard) and its impacts on the exposed community (vulnerability)^16^.

Initially, four electronic databases (MEDLINE through PubMed, Scopus, Science Direct and Google Scholar) were searched up to December 31; 2015.The search strategy was based on the PubMed database model. The key terms were adopted from Medical Subject Heading (MeSH) when possible; otherwise appropriate key words were selected according to the expert idea. The expert team is including the authors’ team and 3 more volunteer PhD students at Health in Emergencies and Disasters from Tehran University of Medical Sciences with previous field experiences in disaster medicine. Titles and abstracts were searched with the following syntax:

- “Site selection”[tiab]AND hospital[tiab]

**Study eligibility**

The inclusion criteria were articles that were published in peer-reviewed journals and had site selection criteria for a hospital. Gray literature including conference proceeding papers and thesis were included as well. All non-English articles were excluded.

**Data collection and analysis**

The key terms were searched in the databases separately and all articles were imported to a bibliographic management program (EndNote X3). Duplications were then omitted. The titles and abstracts were evaluated by two authors (MJM and BR) independently; in the cases that the exclusion criteria could not be applied, the full article was reviewed. If there was disagreement about the eligibility of a particular article between MJM and BR, the third author (AA) was asked to adjudicate. The electronic search was conducted from November 2015 to December 2015.

In the next step, the data were extracted out of the full-text of the included articles. This data included the first author’s name, the year of publication, and the first author’s country, the geographical scope, site selection criteria and the method of study. The references of selected articles was hand searched. Finally, the extracted site selection criteria were categorized through a focus group discussion by the expert team which has been described above already. The final results were sent to the expert team for confirmation through e-mails.

**Quality assessment**

A 9-question checklist was produced to assess the quality of the retrieved publications by authors (Table 3). The Quality-related questions investigated the following components: number of applied criteria for HSS, categorization of the applied criteria (Yes or No), source of applied criteria (i.e. the authors themselves, experts idea or review of literature) , GIS based method (Yes or No), explanation of why the analysis method was used (Yes or No), description of the candidate regions for HSS (Yes or No), number of candidate regions for HSS (i.e. the number of regions or a countless sites), discussion about the limitation (Yes or No), and discussion about the generalizability of the study (Yes or No).

The checklist was filled out by two assessors independently (MJM and BR) and “AA” adjudicated when there was disagreement.

## Results

In the first step 41 studies were retrieved through the bibliographic search. After removing seven irrelevant and nine duplicates, 25 were remained. Sifting process left 15 eligible studies that were published up to the end of December 2015, 2 of them were identified from hand searching of references of included articles. Figure 1 illustrates the related PRISMA flow diagram. The result of quality assessment is summarized in Supporting Information file, S1 Table.

**Figure:**
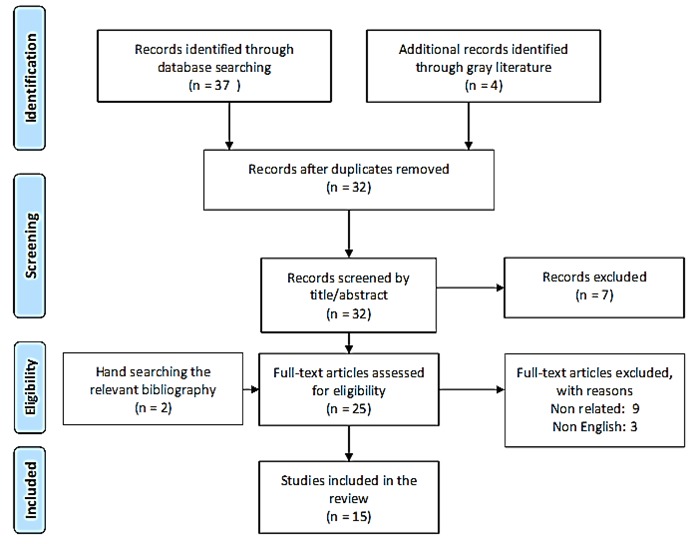
**Fig. 1:** PRISMA Flow diagram for systematic review of hospital site selection criteria

Table 1 summarizes the main results of the present study. The first article was published in 1995^17^. The maximum number of articles were published in 2013, i.e., 6 articles. The total number of authors of the included articles was 38 (2.7 authors per article, in average). The first/corresponding authors were affiliated with different universities; among these, only K.N.Toosi University of Technology (in Iran) was associated with two publications^2^^,^^18^.

**Figure:**
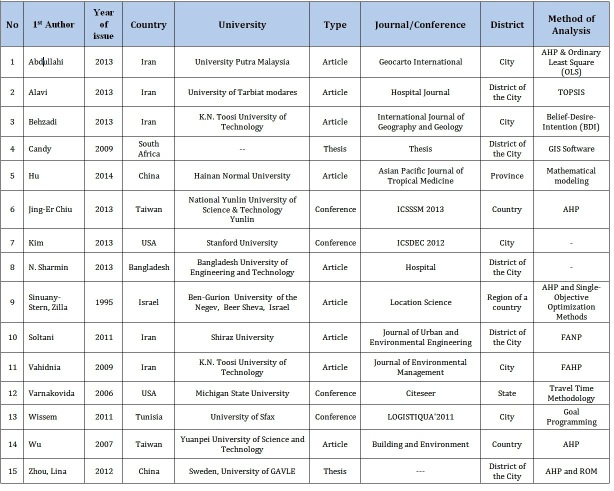
**Table 1:** Main results of the systematic review about disaster risk criteria for hospital site selection

HSS studies performed in China were 2, Iran (5), USA (2), Taiwan (2), Tunisia, South Africa, Israel and Bangladesh (1). Regarding the geographical scope, three studies were performed at the provincial or state level, two at the country level, five at the city level, and five at a district of a city.

With regard to the methodology, 10 articles used Geographical Information System (GIS), 5 studies applied AHP, and one used fuzzy AHP for site selection^19^. One study combined the AHP with the Ordinary Least Squares (OLS) method^18^ while another one combined AHP with the Rank Order Method (ROM)^20^. Other models that were used included mathematical modeling, Belief-Desire-Intention (BDI)^2^, Technique for Order of Preference by Similarity to Ideal Solution TOPSIS^1^, goal programming^11^, fuzzy Analytical Network Process (ANP)^7^, and travel-time methodology^21^.

Through a focus group discussion, with the above described expert team, all 39 HSS criteria were classified into six main groups namely cost, demand, environmental, administrative, disaster risk, and other concerns. The application percentage of each of these groups was 100, 93.3, 53.3, 33.3, 20.0, and 13.3 respectively (Figure 2).

**Figure:**
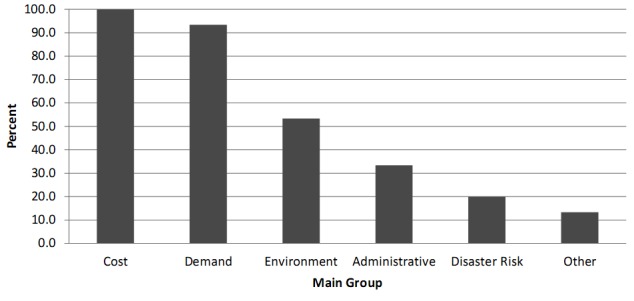
**Fig. 2:** Usage of main groups of Hospital Site Selection Criteria in included articles.

The six mentioned groups were subsequently divided into 16 subgroups (Table 2).

**Figure:**
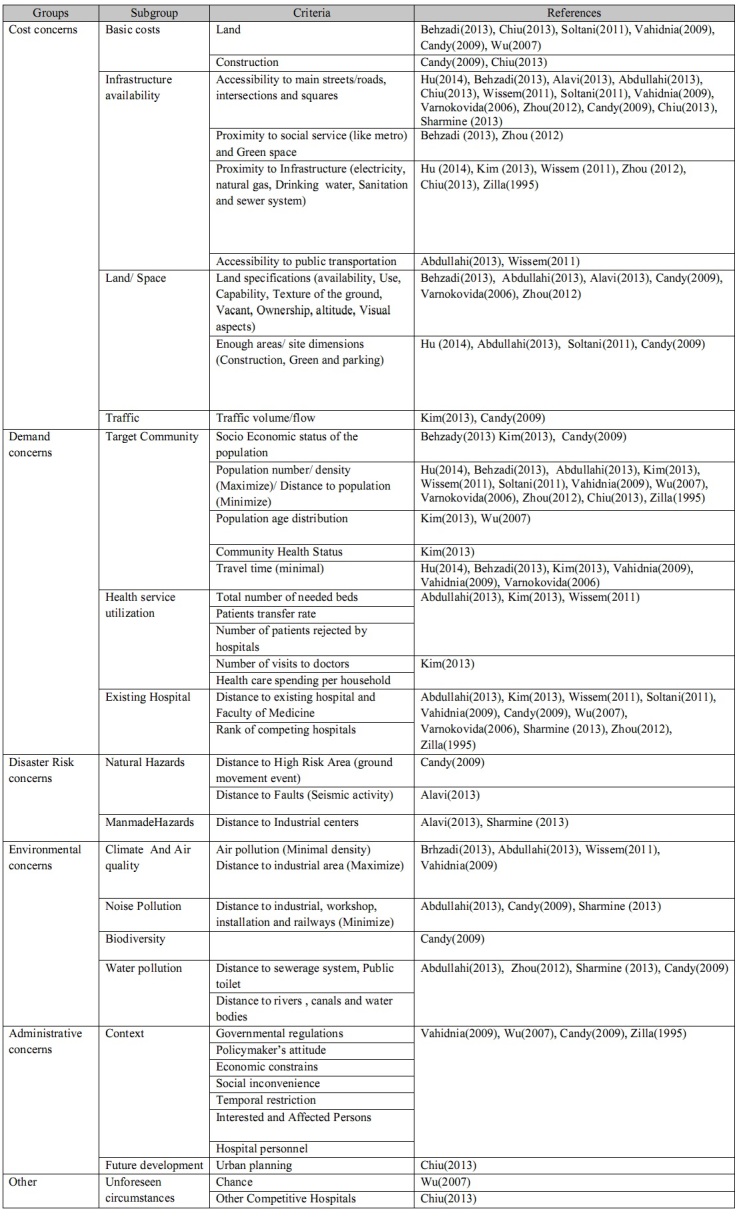
**Table 2:** List of classified criteria for hospital site selection in included articles

## Discussion

This systematic review was conducted to find out the disaster risk related criteria for HSS. According to the results, despite the increasing trend of worldwide disasters, risk related criteria are not taken into consideration to the extent that they should.

It is not mandatory and also not realistic to apply all these factors for HSS. Depending on the strategy for building medical centers, the planners may consider only some of these. For example, Kim et al (2013) conducted HSS in the construction of a hospital for the aging population and considered factors that looked at the real health requirements of the target group; thus, some criteria such as environmental issues (air and sound pollution and sewerage system) were not considered^22^. Soltani et al (2011) used several criteria such as urban planning, traffic volume, and travel time for site selection of a hospital in district five of Shiraz, Iran; however they did not consider environmental and land specification issues in their study^7^. As the main goal of Wu et al (2007) was ensuring competitive advantage for HSS, they considered administrative criteria such as regulations, policymaker’s attitude, and even demand for the hospital personnel. They did not consider environmental concerns and accessibility to infrastructures such as main roads, as well^23^.

Alavi et al. (2013) considered two groups of factors that affect accessibility (roads and social services) and also distance from potential hazards (faults and industrial centers) for one region of the capital city of Tehran, Iran. However they did not use future planning development, real demand for hospital in the region, existing health services, and other land specifications^1^. Also Jing-Er Chiu and Hang-Hao Tsai (2013) used MCDM to determine the optimal location for expansion of a regional teaching hospital in Yunlin County, Taiwan. They used highly detailed criteria including the demand for medical service, cost, transportation, sector support, and future development. The main theme considered in their study was increasing the competitive advantage for the hospital^24^.

In 2014, Xiao-Hua Hu et al developed a model to identify the proper location for medical and health services in a large group of islands in Hainan Province in China. This model was based on minimizing the travel distance for clients of these services. In this study, the real demand and environmental issues were not considered^25^.

To achieve sustainable development, a community should consider important issues in building and utilizing a new hospital. In HSS procedures, environmental considerations are important issues^26^^,^^27^^,^^28^. While important, the majority of publications devoted more attention to cost and demand rather than environmental issues such as air and noise pollution as these have sever negative effects on hospital functions. In addition, following the completion of the hospital, these types of pollution would be aggravated.

Regarding the cost concerns, most of the studies devoted attention to accessibility by main roads and arteries. Other cost subgroups were proximity to infrastructures, and land specifications (availability, use, texture of the ground, being vacant, and ownership). However, none of the articles discussed the beneficial aspect of the proximity of the hospital location to airports or seaports.

Concerning the demand category, health service utilization of the community was assessed according to the total number of required beds, the patient transfer rate, and the number of patients rejected by hospitals^11^^,^^18^^,^^22^. In this category, epidemiological indices such as “burden of disease” and forecasting demand for health care based on demographic factors, economic growth in the area, and even new technologies in health system could be considered. For example, oil and energy activities are a great source of economic development but require a certain type of health care infrastructure when it comes to emergency medical services including trauma and cardiac patients.

As construction of a hospital is a kind of investment, the investors wait for future profit. Hence, threats to this investment should be taken into account. For more than 25 years, WHO has promoted and supported the efforts to the purpose of safe hospitals to improve the function of hospitals in emergencies and disasters. Besides, in Sendai Framework (2015-2030)^10^ the application of the principles of universal design and standardization of building materials in critical facilities such as hospitals is considered with the aim of disaster risk prevention and reduction. Unfortunately, few articles discussed hazards such as faults and industrial areas 1^,^^11^^,^^27^. Other potential hazards, such as floods and man-made disasters, should be considered for at-risk areas. Considering the “risk”, rather than merely hazards, is highly recommended in future permanent and field hospital site selection studies.

The two unclassified criteria, namely unforeseen circumstances and other competitive hospitals were considered in the “Other” category. It is recommended that the safety and security of the candidate locations, rural versus urban areas, local investors, accessibility of communication systems, and availability of competent and qualified staff be considered in this category in further studies.

## Conclusions

Despite the critical role of hospitals in health service delivery in disasters and emergencies and the effect of hospital location on the quality of these services, few articles have considered hazards as the criteria for hospital site selection (HSS). Cost and demand are two groups of criteria that have been addressed more frequently in HSS studies. The decision makers should prospectively match the main objectives of hospital building with the HSS criteria according to the strategy of site selection and the availability of data and resources. Undoubtedly, being safe and remaining functional in emergencies and disasters should be one of the main objectives in HSS in line with Sendai Framework. More comprehensive criteria like “risk of hazards” and “burden of diseases” are suggested to be considered in future studies.

## Limitations

Non-English articles were not included in this study.

## Supporting Information

**Figure:**
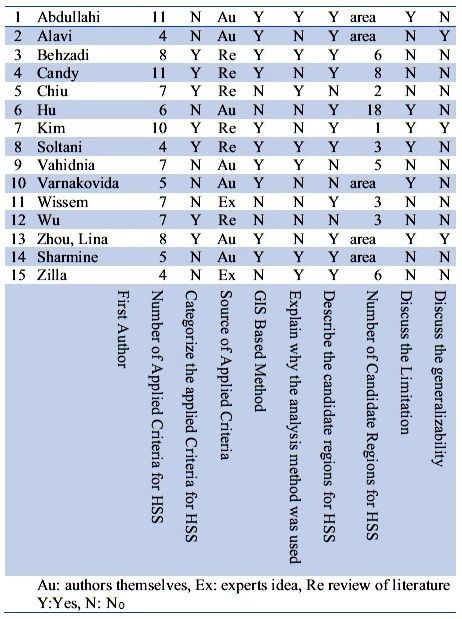
**S1 Table:** Quality assessment results for included publication in HSS systematic review

## Corresponding Author

Ali Ardalan, MD, PhD

E-mail: aardalan@tums.ac.ir

Tehran University of Medical Sciences, Tehran, Iran

## Data Availability

All relevant data are in the article.

## Competing Interests

The authors have declared that no competing interests exist.
